# Distinctive receptor binding properties of the surface glycoprotein of a natural Feline Leukemia Virus isolate with unusual disease spectrum

**DOI:** 10.1186/1742-4690-8-35

**Published:** 2011-05-13

**Authors:** Lisa L Bolin, Chandtip Chandhasin, Patricia A Lobelle-Rich, Lorraine M Albritton, Laura S Levy

**Affiliations:** 1 Department of Microbiology and Immunology and Tulane Cancer Center, Tulane University School of Medicine, 1430 Tulane Avenue SL-38, New Orleans, LA, 70112, USA; 2 Department of Microbiology, Immunology & Biochemistry, University of Tennessee Health Science Center, 858 Madison Avenue, Memphis, TN, 38163, USA; 3 Christiana Hospital, 4755 Ogletown-Stanton Road, Newark, DE 19718, USA

## Abstract

**Background:**

Feline leukemia virus (FeLV)-945, a member of the FeLV-A subgroup, was previously isolated from a cohort of naturally infected cats. An unusual multicentric lymphoma of non-T-cell origin was observed in natural and experimental infection with FeLV-945. Previous studies implicated the FeLV-945 surface glycoprotein (SU) as a determinant of disease outcome by an as yet unknown mechanism. The present studies demonstrate that FeLV-945 SU confers distinctive properties of binding to the cell surface receptor.

**Results:**

Virions bearing the FeLV-945 Env protein were observed to bind the cell surface receptor with significantly increased efficiency, as was soluble FeLV-945 SU protein, as compared to the corresponding virions or soluble protein from a prototype FeLV-A isolate. SU proteins cloned from other cohort isolates exhibited increased binding efficiency comparable to or greater than FeLV-945 SU. Mutational analysis implicated a domain containing variable region B (VRB) to be the major determinant of increased receptor binding, and identified a single residue, valine 186, to be responsible for the effect.

**Conclusions:**

The FeLV-945 SU protein binds its cell surface receptor, feTHTR1, with significantly greater efficiency than does that of prototype FeLV-A (FeLV-A/61E) when present on the surface of virus particles or in soluble form, demonstrating a 2-fold difference in the relative dissociation constant. The results implicate a single residue, valine 186, as the major determinant of increased binding affinity. Computational modeling suggests a molecular mechanism by which residue 186 interacts with the receptor-binding domain through residue glutamine 110 to effect increased binding affinity. Through its increased receptor binding affinity, FeLV-945 SU might function in pathogenesis by increasing the rate of virus entry and spread *in vivo*, or by facilitating entry into a novel target cell with a low receptor density.

## Background

Feline leukemia virus (FeLV) is a naturally occurring gammaretrovirus that infects domestic cats. The outcome of FeLV infection is variable, including malignant, proliferative and degenerative diseases of lymphoid, myeloid and erythroid origin. Determinants of disease outcome are not well understood, but likely involve both viral and host factors. FeLV, like other natural retroviruses, does not occur as a single genomic species but as a closely related, genetically complex family. Sequence variation among natural isolates occurs most commonly in the viral long terminal repeat (LTR) and in the surface-exposed envelope glycoprotein (SU) [1,2]. An unusual natural isolate, designated FeLV-945, was previously identified as the predominant isolate in a geographic and temporal cohort of naturally infected cats [3,4]. The predominant disease presentation in the cohort was a multicentric lymphoma of non-T-cell origin detected in twelve cases, one of which was the original source of FeLV-945. The cohort also included four cases of thymic lymphoma, one case of mast cell leukemia, two cases of myeloproliferative disease and two cases of anemia [3-5]. FeLV-945 has been classified as a member of the FeLV-A subgroup, based on host range and analysis of superinfection interference and on sequence similarity of the envelope protein [3,6]. Members of FeLV-A are ecotropic in host range and utilize feTHTR1, a thiamine transporter on the target cell surface, as a receptor for entry [7].

FeLV-945 differs in sequence from a prototype member of FeLV subgroup A, FeLV-A/61E, in the LTR and in the SU gene [3,6,8,9]. Infection with 61E/945L, a mutant in which the FeLV-945 LTR was substituted for that of FeLV-A/61E, resulted in the relatively rapid induction of thymic lymphoma of T-cell origin. Thus, introduction of the FeLV-945 LTR induced the same tumor as FeLV-A/61E, but did so more rapidly [9]. By contrast, infection with 61E/945SL, a mutant in which both the FeLV-945 LTR and SU gene were substituted for those of FeLV-A/61E, resulted in the rapid induction of multicentric lymphoma of B-cell origin, thus recapitulating the predominant disease detected in the natural cohort [9]. Taken together, these findings implicated the FeLV-945 LTR as a determinant of the rate of disease induction, and FeLV-945 SU as the determinant of disease spectrum. The mechanism by which FeLV-945 SU might influence disease outcome is not known.

As the receptor-binding protein of the virus, natural variation in SU is associated with significant functional impact on receptor utilization, thereby influencing cell tropism, rate of spread, and disease outcome [1,2,10-14]. The FeLV SU protein, analogous to the closely related murine leukemia viruses, contains two amino-terminal hypervariable regions, designated variable region A (VRA) and variable region B (VRB), that comprise the receptor binding domain [1]. Previous work has demonstrated that the VRA domain is the primary determinant of receptor interaction and is sufficient for receptor binding, while the VRB domain is necessary for efficient infection [15-21]. Secondary determinants for receptor binding have also been identified in the carboxy-terminal region of SU and in a central proline-rich region (PRR) known to mediate conformational changes required for virus entry [17,22-24]. FeLV-945 SU differs from that of FeLV-A/61E to a larger extent than other known FeLV-A isolates differ among themselves [3]. Point mutations in FeLV-945 SU, relative to FeLV-A/61E, are largely contained within protein domains having roles in receptor recognition and entry [3,6].

In the present study, unique properties of FeLV-945 SU were characterized that may play a role in its ability to direct disease outcome. Target cell receptor binding was compared between the FeLV-945 and FeLV-A/61E SU proteins. FeLV-945 SU was shown to exhibit an increased efficiency of receptor binding as compared to FeLV-A/61E using a variety of experimental conditions, both when presented in virus particles and in soluble form. The SU proteins of other isolates from the cohort were also found to exhibit an increase in receptor binding efficiency that was comparable to or greater than that observed with FeLV-945 SU. Mutational analyses implicated a region containing the VRB domain of FeLV-945 SU as the major determinant of the distinctive receptor-binding phenotype, and identified a single amino acid residue as primarily responsible for the effect.

## Results

### Relative binding activity of virus particles bearing FeLV-945 Env and of soluble SU proteins

**Figure 1:**
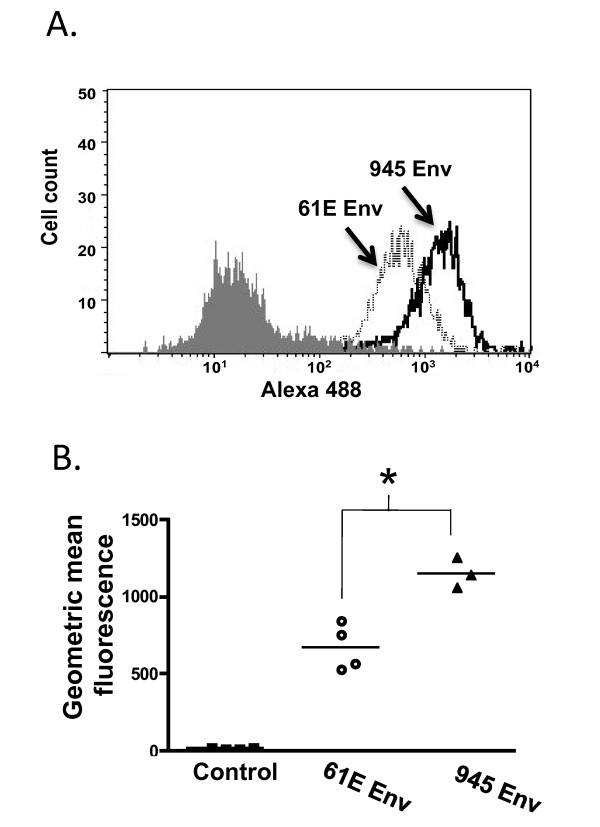
**Comparative binding assays of virus particles bearing the Env protein of FeLV-A/61E or of FeLV-945**. **A**. Feline 3201 cells were incubated with equivalent numbers of virus particles bearing the envelope protein of FeLV-A/61E (61E Env) or FeLV-945 (945 Env), followed by incubation with monoclonal antibody C11D8 to detect the surface-bound viral SU protein and then with an Alexa Fluor 488-conjugated secondary antibody. Virus binding was analyzed by flow cytometry. A representative histogram is shown, demonstrating the binding activity of the particles as indicated and a negative control in which no virus was included in the assay (shaded). **B**. The geometric mean fluorescence of quadruplicate samples from individual assays is indicated, as is the mean of replicate experiments (horizontal bar). Asterisk indicates statistical significance (*; p < 0.001).

**Figure 2:**
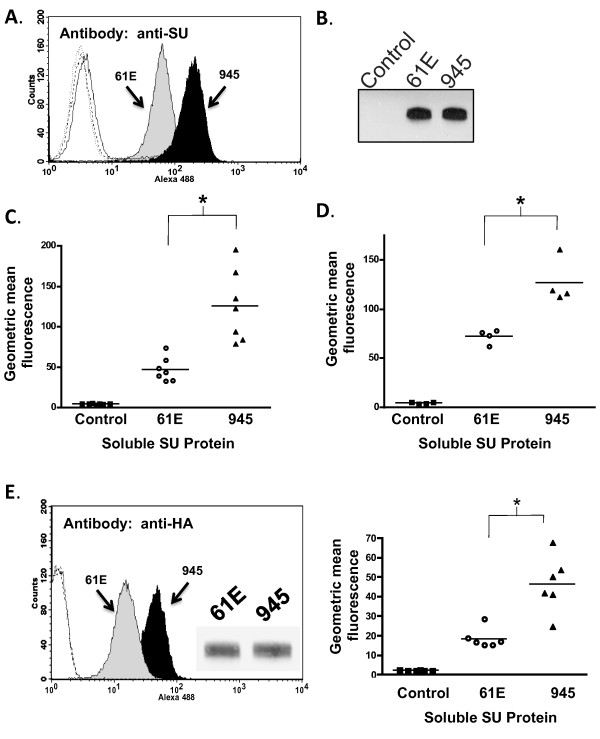
**Comparative binding assays of soluble SU proteins of FeLV-A/61E or FeLV-945**. **A**. A representative histogram is shown from a comparative flow cytometric binding assay demonstrating the binding activity of FeLV-A/61E SU (61E; gray shaded) or FeLV-945 SU (945; black shaded). Soluble SU proteins were quantified precisely using anti-SU antibody C11D8. Feline 3201 cells were incubated with equivalent mass amounts of either SU protein for one hour, followed by incubation with C11D8 antibody to detect the surface-bound viral SU proteins and then with an Alexa Fluor 488-conjugated secondary antibody. Negative controls (open histograms) included cell supernatants of transfections with the empty expression vector, pCS2/Ctrl, and each SU with isotype control antibody. **B**. Chemiluminescent western blot analysis of equivalent mass amounts of FeLV-A/61E and FeLV-945 SU proteins using C11D8 antibody as probe is shown to validate the precision of the infrared quantification. Negative control was supernatants of cells transfected with pCS2/Ctrl. **C - D**. Geometric mean fluorescence of replicate binding assays performed using four independently generated and quantified batches of FeLV-A/61E and FeLV-945 SU protein on either feline 3201 cells (**C**) or murine MDTF/H2 cells (**D**) which express the FeLV-A receptor. Supernatant of mock- or pCS2/Ctrl-transfected cells were used as a negative control. The mean of replicate experiments is represented (horizontal bar). Asterisk indicates statistical significance (*; p < 0.001). **E**. Flow cytometric binding assays performed exactly as in (A) except that analysis was performed using an antibody to detect the HA tag at the C-terminus of soluble SU proteins. Shown are a representative histogram (**left**), anti-HA chemiluminescent western blot analysis of equivalent mass amounts of SU proteins to validate quantification (**inset**), and geometric mean fluorescence of replicate binding assays (**right; **p < 0.001). Negative controls included either SU protein with isotype control antibody (open histograms).

**Figure 3:**
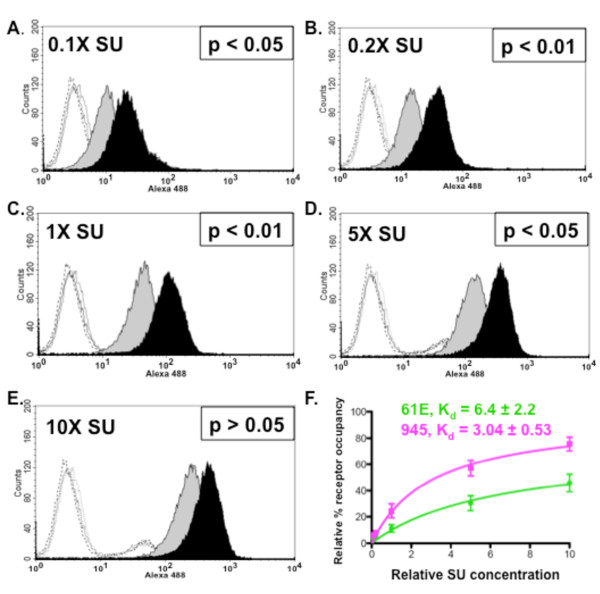
**Increased binding activity of FeLV-945 SU is observed over a 100-fold range of SU concentration**. **A. - E**. FeLV-945 SU or FeLV-A/61E SU proteins in equivalent mass amounts over a 100-fold range (0.1X - 10X) were incubated with feline 3201 cells and processed for flow cytometric binding assays as described in Figure 2. Representative histograms are shown, demonstrating the binding activity of FeLV-A/61E SU (gray shaded) or FeLV-945 SU (black shaded). Negative controls (open histograms) included supernatants from mock-transfected cells (solid line), FeLV-A/61E SU with isotype control antibody (dotted line), and FeLV-945 SU with isotype control antibody (dashed line). Indicated at each SU concentration is the result of statistical analysis of replicate binding assays using four independently generated and infrared-quantified batches of SU proteins. A statistically significant increase in geometric mean fluorescence for FeLV-945 SU binding was considered p < 0.05. **F**. Relative dissociation constants (K_d_) were determined from the data shown in A - E by nonlinear regression analysis using saturation binding equations with an assumption of one site-specific binding (GraphPad Prism5.0).

Flow cytometric binding assays were first performed to assess the relative strength of receptor binding by virus particles bearing the Env protein of FeLV-945 or of prototype FeLV-A/61E. For this purpose, equivalent infectious titer of particles bearing either Env protein were allowed to bind to feline 3201 T-lymphoid cells, after which binding was detected using monoclonal antibody C11D8 directed against FeLV SU. The results demonstrated that virus particles bearing FeLV-945 SU bind to the cell surface receptor significantly more efficiently than do particles bearing the FeLV-A/61E SU (p < 0.001; Figure 1). While these studies suggest differential binding properties of the viruses examined, the experiment as performed cannot account for the possibility that FeLV SU may be present in higher amounts, or may be differentially displayed, on the surface of virus particles in a manner as to influence receptor binding affinity. To control for these possibilities, soluble FeLV-945 and FeLV-A/61E SU proteins were expressed and quantified precisely by western blot analysis using anti-SU antibody C11D8 and an infrared dye-conjugated secondary antibody followed by densitometric analysis. The presence of equivalent mass amounts of protein was then verified visually using chemiluminescent western blot analysis. Having quantified the proteins, equivalent mass amounts were then used in flow cytometric binding assays on feline 3201 T-cells using C11D8 antibody. By this analysis, FeLV-945 SU was observed to bind cell surface receptor with greater efficiency than did FeLV-A/61E SU (Figure 2A-B). Replicate binding assays, using four independently prepared and quantified protein preparations, demonstrated the increased binding of FeLV-945 SU to be statistically significantly higher than that of FeLV-A/61E SU (p < 0.001; Figure 2C). Enhanced binding of FeLV-945 SU relative to FeLV-A/61E was also observed on other feline cells lines including FEA and AH927 cells (data not shown). Further, a statistically significant increase in cell surface receptor binding was observed on MDTF/H2 [25], a mouse cell line engineered to express the FeLV-A receptor (p < 0.001; Figure 2D). C11D8, the monoclonal antibody used to detect SU binding in the assays described above, recognizes an epitope conserved between FeLV-A/61E and FeLV-945 SU proteins [26]. To further confirm the enhanced cell surface binding phenotype of FeLV-945 SU, binding assays were performed using an antibody that recognizes the HA epitope tag fused to the C-terminus of the soluble SU proteins. This measure also demonstrated the binding of FeLV-945 SU to be statistically significantly greater than that of FeLV-A/61E SU (p < 0.001; Figure 2E). To determine whether the increased receptor binding of FeLV-945 SU could be observed over a broad range of protein concentrations, binding assays were performed using FeLV-A/61E or FeLV-945 SU in equivalent mass amounts varying over a 100-fold range. A statistically significant increase in binding activity of FeLV-945 SU was observed at each concentration tested except at the highest amount (Figure 3A - E). Nonlinear regression analysis of the results using saturation binding equations revealed a 2-fold difference in dissociation constant (K_d_; Figure 3F).

**Figure 4:**
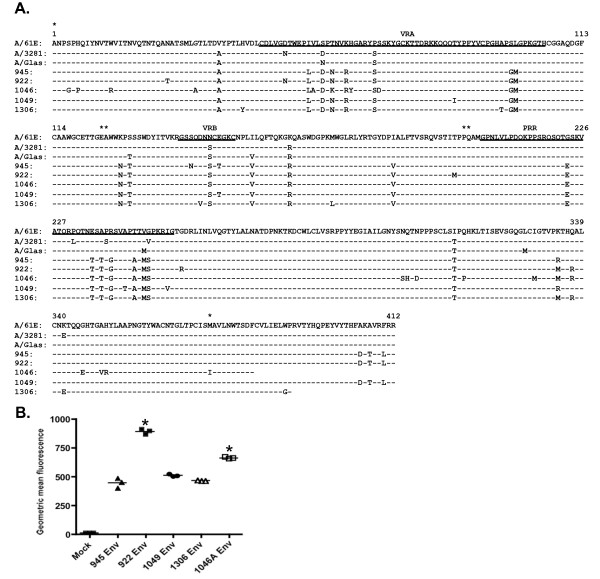
**Pseudotype virus particles bearing the Env protein from other cohort isolates exhibit binding properties equivalent to, or significantly greater than, FeLV-945**. **A**. Sequence comparison of SU proteins from prototype FeLV-A isolates FeLV-A/61E [GenBank:AAA93093], FeLV-A/3281 [GenBank:AAA43051] and FeLV-A/Glasgow [GenBank:AAA43053], from FeLV-945 [GenBank:AAT76450] and from other representatives of the cohort. FeLV-922 [GenBank:AAT76452], FeLV-1046A [GenBank:AAT76457] and FeLV-1049 [GenBank:AAT76458] were isolated from multicentric lymphomas. FeLV-1306 [GenBank:AAT76463] was isolated from myeloproliferative disease. Indicated is the complete amino acid sequence of the mature SU protein encoded by each isolate. The sequence encoded by FeLV-A/61E is shown, identity to FeLV-A/61E is indicated (-), as are amino acid substitutions by one-letter code. The positions of previously identified functional domains VRA, VRB and PRR are underlined. Asterisks indicate positions of the regions used to create substitution mutants shown in Figure 6A and described in the text. **B**. Flow cytometric binding assays were performed as in Figure 1 except using equivalent titers of pseudotyped viral particles bearing the envelope proteins (Env) of FeLV-945, FeLV-922, FeLV-1049, FeLV-1306, or FeLV-1046A. The geometric mean fluorescence from individual assays is shown, as is the mean of three independent replicate experiments (horizontal bars). Asterisk indicates statistical significance (*; p < 0.001).

As described above, FeLV-945 is a representative isolate from a natural cohort of infected animals in which the predominant disease presentation was a distinctive multicentric lymphoma of non-T-cell origin [3-5]. In previous studies, proviral DNA was amplified by PCR from several cases of multicentric lymphoma (945, 922, 1046, 1049) and from a case of myeloproliferative disease (1306). Sequence analysis of the SU genes demonstrated close relatedness but not identity to FeLV-945, although host range and superinfection interference analysis demonstrated a phenotype consistent with FeLV subgroup A [6]. Sequence comparison demonstrated a set of residues in common among isolates from the cohort that are distinct from previously characterized SU proteins from subgroup A members FeLV-A/61E, FeLV-A/3281 and FeLV-A/Glasgow. The latter are nearly identical to each other despite having been isolated from distant geographic locations over a period of many years [27], but are clearly distinct from the cohort isolates within the functional domains of SU (Figure 4A). To examine whether the observed commonalities in SU sequence confer the increased receptor binding activity typical of FeLV-945 on other isolates from similar disease outcome, pseudotype particles bearing Env proteins from FeLV-945, FeLV-922, FeLV-1049, FeLV-1306, and FeLV-1046A [6] were used for flow cytometric binding assays on feline 3201 T-cells. The results demonstrated cell surface receptor binding activity comparable to or significantly greater than that of pseudotype particles bearing FeLV-945 Env. Receptor binding by FeLV-922 or FeLV-1046A Env pseudotypes was significantly increased as compared to pseudotypes bearing the other Env proteins examined (p < 0.001; Figure 4B).

### Mutational analysis does not implicate the consensus VRA domain of FeLV-945 SU as a determinant of binding phenotype

**Figure 5:**
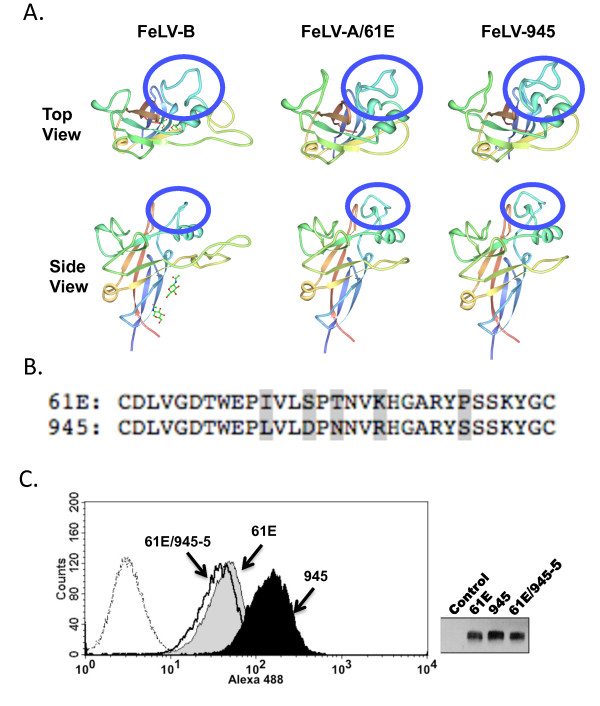
**A loop structure predicted by computational modeling in the VRA domain of FeLV-A is not sufficient to confer the binding phenotype of FeLV-945 SU**. **A**. Ribbon diagram of homology models of the receptor binding domain in FeLV-A/61E and FeLV-945 SU proteins. Homology modeling was performed using the SwissModel Program and the known crystal structure of the receptor binding domain of FeLV-B SU (FeLV-B 1LCS) as a modeling template. A prominent loop (circled) was predicted by the models within the VRA domain of FeLV-A/61E and FeLV-945 proteins, and is distinct from the structure of FeLV-B in the same region. **B**. Comparison of amino acids sequences of FeLV-A/61E and FeLV-945 in the predicted VRA domain loop. The five amino acid differences between the sequences are indicated by shading. **C**. Comparative flow cytometric binding assays of SU proteins encoded by FeLV-A/61E, FeLV-945 and 61E/945-5, a mutant in which the FeLV-945 sequence at all of the five highlighted residues shown in Figure 5B was substituted by site-directed mutagenesis into FeLV-A/61E. Binding assays were performed using feline 3201 cells as described in Figure 2. A representative histogram is shown (left panel), demonstrating the binding activity of FeLV-A/61E SU (gray shaded), FeLV-945 SU (black shaded) and 61E/945-5 SU (open histogram, solid line). Negative controls (open histograms, broken lines) include supernatants from pCS2/Ctrl-transfected cells and 61E/945-5 SU with isotype control antibody. Right panel shows chemiluminescent western blot analysis to validate equivalent mass amounts of the SU proteins used in the binding assay as previously quantified by infrared dye-based densitometry.

To identify the domain(s) within FeLV-945 SU responsible for the increased binding affinity, we first considered VRA since that domain has been previously identified as the major determinant of receptor interaction in murine and feline gammaretroviral SU proteins [15-21]. We began by examining the predicted crystal structure of FeLV-945 VRA to identify potential areas of interest as compared to prototype FeLV-A. Crystal structure of the receptor-binding domain of FeLV subgroup B SU has been previously described [28], although no such structure has yet been described for FeLV-A. Thus, homology modeling of the receptor binding domain in the SU proteins of FeLV-A/61E and FeLV-945 was performed using the known FeLV-B SU structure [28] as a modeling template for the SwissModel Program [29-31] (Figure 5A). Computational models thereby generated predict a prominent loop in the VRA domain of both FeLV-A/61E and FeLV-945 SU that is distinct in structure from FeLV-B and is predicted to protrude on the receptor-binding surface (Figure 5A). The predicted structure is a cysteine-delimited loop of 31 residues that appears similar in conformation in FeLV-945 and FeLV-A/61E. However, the loop sequence includes five residues that diverge between FeLV-945 and FeLV-A/61E, thereby implicating the divergent residues in the differing receptor binding phenotypes of the FeLV-945 and FeLV-A/61E SU proteins (Figure 5B). To test the hypothesis that the FeLV-945 sequence in the predicted VRA domain loop confers increased binding efficiency, site-directed mutagenesis was utilized to replace the five divergent residues in the sequence of FeLV-A/61E SU with those of FeLV-945, yielding a mutant SU gene designated 61E/945-5. Soluble SU expressed by 61E/945-5 was then prepared and quantified for use in comparative binding assays with SU proteins from FeLV-945 and FeLV-A/61E. The results demonstrated that the binding phenotype of the 61E/945-5 mutant SU is statistically indistinguishable from that of the FeLV-A/61E parent protein (Figure 5C, left). Equivalent mass amounts of each protein were used in the binding assay as confirmed by quantitative western blot analysis (Figure 5C, right). The reciprocal mutant, 945/61E-5, was also constructed to replace the five divergent residues in the sequence of FeLV-945 SU with those of FeLV-A/61E. Soluble SU expressed by 945/61E-5 was precisely quantified and used in comparative binding assays. The results demonstrated the binding phenotype of the 945/61E-5 mutant to be statistically indistinguishable from that of FeLV-945 SU (data not shown).

**Figure 6:**
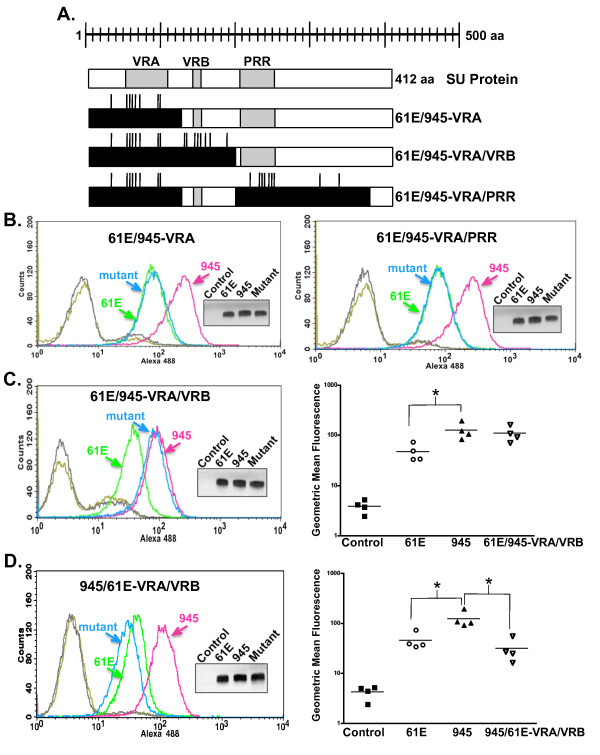
**Substitution of both FeLV-945 VRA and VRB is sufficient to confer the enhanced binding phenotype to FeLV-A/61E SU**. **A**. Diagram of the 412-amino acid FeLV-A/61E SU protein and mutants into which FeLV-945 sequences were substituted. Positions of the VRA, VRB, and PRR domains are indicated (shaded boxes). FeLV-945 sequences that have been substituted into FeLV-A/61E SU to construct each mutant are indicated (black boxes), and vertical lines represent the relative locations of amino acid sequence differences between the two SU proteins. **B. - D**. Comparative flow cytometric binding assays of SU proteins encoded by FeLV-A/61E, FeLV-945 and substitution mutant SU proteins as indicated. Binding assays were performed using feline 3201 cells as described in Figure 2. Representative histograms are shown, demonstrating the binding activity of FeLV-A/61E SU (green), FeLV-945 SU (pink) and the substitution mutant indicated in each case (blue). Negative controls included supernatants from pCS2/Ctrl-transfected or mock-transfected cells (gray) and each mutant SU with isotype control antibody (gold). Inset in each panel shows chemiluminescent western blot analysis to validate equivalent mass amounts of the SU proteins used in the binding assay as previously quantified by infrared dye-based densitometry. **C. and D., Right panels**. Replicate binding assays were performed using four (61E/945-VRA/VRB) or two (945/61E-VRA/VRB) independently generated and infrared-quantified batches of SU proteins. The geometric mean fluorescence from individual assays is shown, as is the mean of four independent replicate experiments (horizontal bar). Asterisk indicates statistical significance (*; **C**: p < 0.05; **D**: p < 0.01).

Having determined that the divergent residues within consensus VRA do not determine receptor-binding affinity, a more comprehensive region surrounding VRA was then examined through the use of substitution mutants. Segments of the FeLV-A/61E SU gene were replaced with corresponding segments of FeLV-945 SU so that the resultant proteins would be substituted of either a VRA domain-containing region or both VRA- and PRR-containing regions (61E/945-VRA or 61E/945-VRA/PRR respectively, Figure 6A). Specifically, the substituted VRA-containing region included 124 residues from alanine at position 1 to glutamic acid at position 124. The substituted PRR-containing region included 172 residues from glutamine at position 202 to methionine at position 373 (Figure 4A). After substitution of each region from FeLV-945 into FeLV-A/61E, soluble SU proteins were then expressed from each mutant and quantified precisely using infrared dye-based densitometric analysis of western blots. Equivalent mass amounts of protein were used in comparative binding assays as verified visually using chemiluminescent western blot analysis. The resulting binding assays demonstrated a phenotype for 61E/945-VRA and 61E/945-VRA/PRR that was identical to the FeLV-A/61E parent SU protein (Figure 6B). Thus, analysis of point mutations and substitutions indicates that consensus VRA is not a major determinant of FeLV-945 binding phenotype.

### Substitutional analysis implicates a VRB-containing region as the major determinant of the binding phenotype

**Figure 7:**
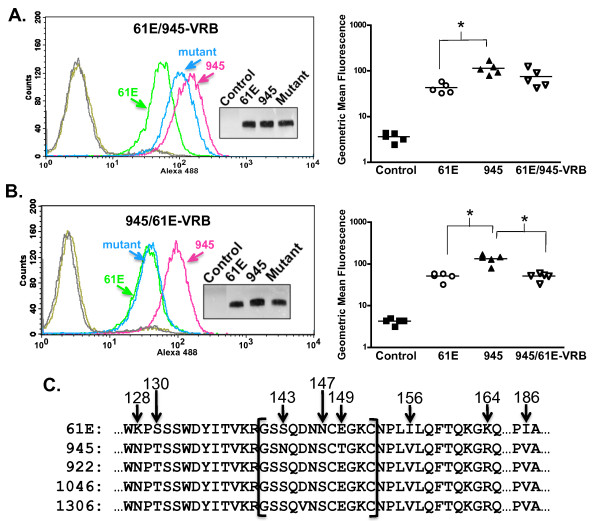
**FeLV-945 VRB is sufficient to confer the enhanced binding phenotype to FeLV-A/61E SU**. **A - B**. Comparative flow cytometric binding assays of SU proteins encoded by FeLV-A/61E, FeLV-945 and 61E/945-VRB (in **A., left**) or the reciprocal mutant, 945/61E-VRB (in **B., left**). Binding assays were performed using feline 3201 cells as described in Figure 2. Representative histograms are shown, demonstrating the binding activity of FeLV-A/61E SU (green), FeLV-945 SU (pink) and the substitution mutant indicated in each case (blue). Negative controls included supernatants from pCS2/Ctrl-transfected cells (gray) and each mutant SU with isotype control antibody (gold). **Inset **in each panel shows chemiluminescent western blot analysis to validate equivalent mass amounts of the SU proteins used in the binding assay as previously quantified by infrared dye-based densitometry. Replicate binding assays were performed (**right panels**) using two (945/61E-VRB), three (61E/945-VRB) or five (FeLV-A/61E and FeLV-945) independently generated and titered batches of SU proteins. The geometric mean fluorescence from individual assays is shown, as is the mean of five independent replicate experiments (horizontal bar). Asterisk indicates statistical significance (*; **A**: p < 0.01; **B**: p < 0.001). **C**. Amino acid sequence of the VRB-containing domain of FeLV/A-61E, compared to that of FeLV-945 and other cohort isolates (FeLV-922, FeLV-1046A, FeLV-1306). Indicated by brackets is consensus VRB, and sequence differences are indicated by the amino acid position number within the mature SU protein.

A region of SU containing the VRB domain was next examined for contribution to the FeLV-945 binding phenotype. First, a substitution mutant was constructed in which both VRA- and VRB-containing regions of FeLV-A/61E were substituted with those of FeLV-945 (61E/945-VRA/VRB; Figure 6A). The substituted VRB-containing region included 77 residues from alanine at position 125 to proline at position 201 (Figure 4A). Comparative binding assays using equivalent mass amounts of protein demonstrated the binding phenotype of 61E/945-VRA/VRB SU to be nearly identical to FeLV-945 SU (Figure 6C), thus implicating the VRB domain. The reciprocal mutant 945/61E-VRA/VRB was then constructed, in which FeLV-A/61E VRA and VRB regions were substituted for those of FeLV-945. Consistent with the implication of VRB as the relevant determinant, comparative binding assays demonstrated a phenotype of the 945/61E-VRA/VRB mutant that was similar to FeLV-A/61E SU and significantly decreased when compared to FeLV-945 SU (p < 0.01; Figure 6D). Studies were next performed to delineate whether the VRB domain-containing region alone was sufficient to determine the binding phenotype. A mutant was constructed in which the VRB-containing region of FeLV-945 alone was substituted into FeLV-A/61E SU to construct a mutant designated 61E/945-VRB. Comparative binding assays using equivalent mass amounts of protein demonstrated the binding phenotype of 61E/945-VRB SU to be similar to FeLV-945 SU although the increased binding relative to FeLV-A/61E did not reach statistical significance (Figure 7A). A reciprocal mutant was constructed, designated 945/61E-VRB, in which the VRB-containing region of FeLV-A/61E was substituted into that of FeLV-945. Comparative binding assays using equivalent mass amounts of protein demonstrated a binding phenotype for 945/61E-VRB SU that was statistically indistinguishable from that of FeLV-A/61E SU and significantly different from FeLV-945 SU (p < 0.001; Figure 7B). These results implicate the 77-amino acid VRB-containing segment as largely responsible for the increased binding efficiency of FeLV-945 SU. The VRB-containing segment exchanged in these studies includes eight amino acid sequence differences between FeLV-945 and FeLV-A/61E, three of which (positions 143, 147, and 149) are localized within consensus VRB (Figure 7C). Two of the differences, at positions 143 and 149, are not shared with other cohort isolates including FeLV-922 and FeLV-1046, whose SU proteins exhibit even more efficient receptor binding than FeLV-945. The asparagine-to-serine change at position 147 is shared among other cohort isolates as are the changes at positions 128, 130, 156, 164 and 186.

### Mutational analysis implicates a single residue as the major determinant of binding phenotype

**Figure 8:**
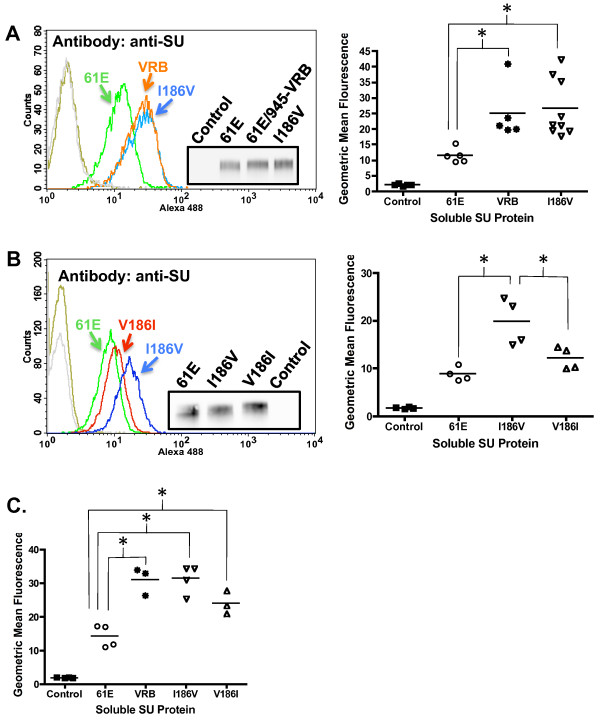
**A single amino acid residue at position 186 accounts for the ability of a VRB-containing domain to confer increased receptor binding affinity to FeLV-A/61E SU**. **A**. A representative histogram from a comparative flow cytometric binding assay of SU proteins encoded by FeLV-A/61E (green), 61E/945-VRB (orange) and I186V (blue) is shown (**left**). Binding assays were performed as described in Figure 2 using anti-SU antibody C11D8. Negative controls include supernatants from pCS2/Ctrl-transfected cells (gray) and I186V SU with isotype control antibody (gold). **Inset **shows chemiluminescent western blot analysis with C11D8 antibody to validate equivalent mass amounts of the SU proteins used in the binding assay. Replicate binding assays were performed (**right**) using six (I186V) or four (FeLV-A/61E and 61E/945-VRB) independently generated and titered batches of SU proteins (p < 0.05). **B**. A representative histogram (**left**) and geometric mean fluorescence of replicate flow cytometric binding assays (**right**) is shown demonstrating the binding activity of SU proteins encoded by FeLV-A/61E (green), I186V (blue) or the reciprocal mutant V186I (red). Negative controls included supernatants from pCS2/Ctrl-transfected cells (gray) and V186I SU with isotype control antibody (gold). Replicate binding assays were performed as in A using four independently generated and titered batches of each SU protein (p < 0.05). **Inset **shows chemiluminescent western blot analysis with C11D8 antibody to validate equivalent mass amounts of the SU proteins used in the binding assay. **C**. Geometric mean fluorescence of replicate binding assays using soluble SU proteins expressed from FeLV-A/61E, 61E/945-VRB, I186V, and V186I is shown (p < 0.05). Negative control is supernatant of pCS2/Ctrl-transfected cells. Comparative flow cytometric binding assays were performed as in A except using anti-HA antibody to detect SU protein. Assays were performed with three (61E/945-VRB) or four (FeLV-A/61E, I186V, V186I) independently generated and titered batches of SU proteins.

To identify the residues within the VRB-containing segment responsible for its influence on binding phenotype, a point mutant was first constructed in which the asparagine at residue 147 of FeLV-A/61E was replaced with serine as appears in FeLV-945 (N147S). Comparative binding assays using equivalent mass amounts of SU proteins demonstrated the binding phenotype of N147S SU to be indistinguishable from FeLV-A/61E (Table 1). A point mutant was then constructed in which the residues at positions 143, 147 and 149 in FeLV-A/61E SU were changed to those of FeLV-945 (VRB3aa). Comparative binding assays using equivalent mass amounts of SU proteins demonstrated the binding phenotype of VRB3aa SU to be indistinguishable from FeLV-A/61E (Table 1). Thus, having identified no residues within consensus VRB as responsible for the binding phenotype, mutational analysis was then performed at positions 128, 130,156,164 and 186 where additional sequence differences were identified. Point mutants were constructed in FeLV-A/61E in which the residues at positions 128 and 130 or 156 and 164 were substituted with those of FeLV-945 (K128N/S130T and I156V/K164R, respectively). Comparative binding assays using equivalent mass amounts of SU proteins demonstrated the binding phenotypes of K128N/S130T and I156V/K164R to be indistinguishable from FeLV-A/61E (Table 1). Only when the isoleucine-to-valine change at position 186 was incorporated into the mutants was the binding phenotype affected. Specifically, the K128N/S130T and I156V/K164R mutants were furthered altered to include the isoleucine-to-valine change at position 186 (K128N/S130T/I186V and I156V/K164R/I186V, respectively). Comparative binding assays using equivalent mass amounts of SU proteins demonstrated that K128N/S130T/I186V and I156V/K164R/I186V SU bound to receptor with increased efficiency and exhibited a binding phenotype indistinguishable from 61E/945-VRB (Table 1). Indeed, a point mutant of FeLV-A/61E SU altered to contain only the isoleucine-to-valine change at residue 186 demonstrated a binding phenotype statistically distinct from FeLV-A/61E SU and equivalent to 61E/945-VRB SU (Figure 8A). A reciprocal mutant, V186I, was constructed in which the isoleucine characteristic of FeLV-A/61E at position 186 was substituted into FeLV-945 SU. Comparative binding assays using equivalent amounts of SU proteins demonstrated a binding phenotype for V186I that was statistically significantly reduced as compared to I186V and indistinguishable from FeLV-A/61E (Figure 8B). To confirm, the binding assays were then repeated using an antibody that recognizes the HA epitope tag fused to the C-terminus of the soluble SU proteins. The results confirmed the key finding that the I186V binding phenotype is statistically indistinguishable from that of 61E/945-VRB and distinct from FeLV-A/61E (Figure 8C). Regarding the reciprocal mutant, V186I, analysis with anti-HA antibody demonstrated reduced binding as compared to I186V but which did not reach the level of statistical significance (Figure 8C). Taken together, these findings do not implicate the consensus VRB domain as the determinant of increased binding affinity, but rather implicate a single residue at position 186 as the major determinant of binding phenotype.

**Table 1:** Summary of comparative flow cytometric binding assays performed using soluble SU proteins of FeLV-A/61E, 61E/945-VRB or mutant FeLV-A/61E SU proteins substituted of specific amino acids within and surrounding the consensus VRB domain of FeLV-945

**Soluble SU Protein**^**a**^	**Average GMF of Replicate Binding Assays**^**b**^	Comparable Binding Phenotype
FeLV-A/61E	12.49	N/A

61E/945-VRB	22.67	N/A

VRB3aa	8.64	FeLV-A/61E

N147S	12.16	FeLV-A/61E

K128N/S130T	11.92	FeLV-A/61E

I156V/K164R	10.09	FeLV-A/61E

K128N/S130T/I186V	22.65	61E/945-VRB

I156V/K164R/I186V	21.69	61E/945-VRB

^**a**^Comparative flow cytometric binding assays were performed using FeLV-A/61E, 61E/945-VRB, or mutated SU proteins named according to the individual FeLV-945 amino acid residues substituted into FeLV-A/61E as shown in Figure 7C. Binding assays were performed using equivalent mass amounts of each SU protein and feline 3201 cells.

^**b**^The average geometric mean fluorescence (GMF) is shown for three (N147S), four (VRB3aa, I156V/K164R, K128N/S130T, I156V/K164R/I186V), or five (K128N/S130T/I186V) replicate binding assays using three or four independently generated and titered batches of SU proteins.

### Computational modeling predicts a mechanism by which residue 186 influences the increased binding affinity of FeLV-945 SU

**Figure 9:**
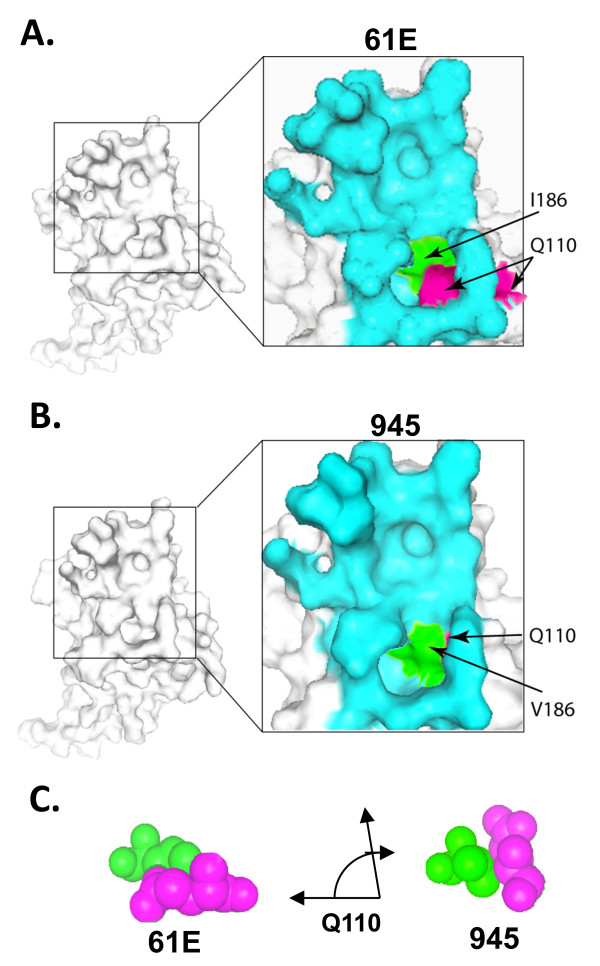
**Structural modeling predicts that residue 186 lies near the end of the putative receptor binding cleft and influences packing of the conserved residue glutamine 110 (Q110)**. **A. - B**. The receptor binding domains of FeLV-A/61E (**A**) and FeLV-945 (**B**) were modeled using SwissModel and the molecular surfaces were calculated and drawn using 3D Molecule Viewer (Vector NTI, Invitrogen). Full views of the predicted surfaces are shown in white and a close up of the putative binding cleft (inset) is shown in cyan with residue 186 shown in green and residue 110 shown in magenta. **C**. Spacefilled depictions of residue 186 (green) and residue 110 (magenta) show the predicted difference in the position of Q110 between the SU of FeLV-A/61E and FeLV-945.

Computational modeling of the putative SU receptor-binding surface demonstrates a change in structure associated with the isoleucine-to-valine change at position 186, and reveals a mechanism that may account for the influence of that change on the binding affinity of FeLV-945 SU (Figure 9). Specifically, modeling predicts that the isoleucine-to-valine change at position 186 affects receptor binding by inducing a major change in the position of an adjacent glutamine residue, Q110, which is conserved in both SU proteins. In FeLV-A/61E SU (Figure 9A), the relatively bulky isoleucine side chain at position 186 effectively pushes Q110 into the lower end of a large binding cleft, thus narrowing the lower end. In FeLV-945 SU (Figure 9B), by comparison, the more compact valine side chain at position 186 is predicted to re-orient Q110 out of the putative binding site at a roughly 90° angle from its position in FeLV-A/61E SU (Figure 9C). The result is that Q110 does not protrude into the lower end of the binding cleft, the predicted consequence of which is that the lower end is twice as wide when valine as compared to isoleucine appears at position 186. The wider receptor-binding site may represent a better surface conformation for interaction of FeLV-945 SU with the receptor residues.

## Discussion

FeLV-945 was isolated from a natural cohort of infected cats in which the predominant disease presentation was a distinctive multicentric lymphoma of non-T-cell origin [3-5]. FeLV-945 was assigned through studies of host range and superinfection interference to FeLV subgroup A [6], although the FeLV-945 LTR and SU gene were shown to contain unique sequence elements [3,6,8]. Indeed, when substituted into FeLV-A prototype isolate, FeLV-A/61E, the unique LTR and SU gene of FeLV-945 were shown to redirect the outcome of infection from T-cell lymphoma of the thymus occurring after prolonged latency to a relatively rapid induction of multicentric lymphoma of B-cell origin, thus recapitulating the natural disease outcome seen with FeLV-945. Further studies demonstrated that FeLV-945 SU is strictly required for the shift in pathogenesis [9]. The mechanism by which FeLV-945 SU acts to determine the outcome of disease induction is not known. In the present study, FeLV-945 SU was observed to bind feline cells, or murine cells expressing the FeLV-A receptor, with significantly increased efficiency as compared to FeLV-A/61E SU, when present either in virus particles (Figure 1) or in soluble form (Figure 2). These findings were documented by flow cytometric binding assay using a monoclonal antibody, C11D8, that recognizes an epitope conserved between FeLV-A/61E and FeLV-945 SU proteins [26]. Thus, the enhanced receptor binding phenotype of FeLV-945 SU is not likely to represent differential binding of the C11D8 antibody. Confirmatory results were obtained using an antibody that recognizes the HA epitope tag fused to the C-terminus of the soluble SU proteins (Figure 2 and 8).

Significantly increased efficiency of receptor binding by soluble FeLV-945 SU was observed using different feline cell lines, including 3201 T-lymphoid cells, AH927 and FEA fibroblast lines (Figure 2 and data not shown). Increased binding was observed over a 100-fold range of SU concentration, with a statistically significant difference observed at all but the highest concentration (Figure 3A - E). These findings indicate that FeLV-945 SU binds receptor with greater affinity, and predicts that FeLV-945 SU would bind the cell surface receptor more efficiently under physiological conditions where the amount of receptor and/or virus may be limiting. By virtue of this phenotype, FeLV-945 SU might then act as a determinant of pathogenesis by increasing the rate of virus entry and spread *in vivo*, or by facilitating entry into a novel target cell with a low receptor density. The analysis further demonstrated a 2-fold difference in dissociation constant (K_d_; Figure 3F), which, while seemingly modest, might have a major impact during an exponentially spreading infection *in vivo*. While we do not yet know the functional impact of increased binding affinity of FeLV-945 SU, it is useful to consider the quantitative modeling of replicative advantage that may be conferred by a mutation in the virus genome. Others have calculated that a newly arising virus mutation which affords a 1% replicative advantage would represent 50% of the virus population within 400 replication cycles [32]. These observations indicate that a replicative advantage (or in this case, increased affinity for receptor-binding) need not be large to impact an exponentially spreading infection.

The cohort from which FeLV-945 was isolated was collected from a single veterinary practice in Pasadena, California over a period of six years [3,4]. Considering the limited geographic and temporal spread of the cohort, the animals were presumably infected by a similar spectrum of natural FeLV isolates circulating among the population. This possibility is supported by the observation that SU genes isolated from other disease cases in the cohort were found to share most of the unique sequence features of FeLV-945 SU, and to exhibit greater sequence identity to each other than to other members of FeLV subgroup A across the functional domains of SU (Figure 4A). This observation is particularly remarkable since other previously described members of FeLV subgroup A demonstrate near identity across SU despite having been isolated from across the world over a period of many years [1,2,27]. Pseudotype virus particles bearing Env protein from cohort isolates were shown to exhibit a receptor-binding phenotype comparable to that of FeLV-945 SU, or in the case of 922 and 1046A pseudotypes, significantly more efficient binding (Figure 4B). These results support the hypothesis that distinctive SU sequences across the functional domains confer the increased binding efficiency characteristic of FeLV-945 SU and other cohort isolates. To test this hypothesis, and to determine which of the functional domains confers the binding phenotype, computational modeling was first performed to examine the potential functional impact of amino acid differences in the receptor-binding domain of FeLV-945 as compared to FeLV-A/61E. While the crystal structure of the receptor-binding domain of FeLV subgroup A is not available, such structure is available for FeLV subgroup B [28]. Using the FeLV-B structure as a modeling template, a cysteine-delimited loop of 31 amino acids was predicted to occur in both FeLV-945 and FeLV-A/61E SU at a position likely to involve the receptor-binding surface (Figure 5A - B). This loop, contained fully within the VRA domain, harbors five amino acid differences between the two sequences (Figure 5B). The possible impact of these changes was of interest, considering the several demonstrations of VRA as the primary determinant of receptor interaction in murine and feline gammaretroviral SU proteins [15-21]. Reciprocal substitution of all five residues between the SU proteins of FeLV-945 and FeLV-A/61E, however, failed to influence the binding phenotype (Figure 5C). These findings indicate that the sequence of the predicted VRA loop is not sufficient to determine the binding phenotype.

To further delineate the responsible domain, substitution mutants were created in which regions containing the VRA, PRR, and VRB domains of FeLV-945 SU and FeLV-A/61E SU were reciprocally replaced (Figure 6A). Comparative binding assays using equivalent mass amounts of soluble SU protein confirmed that substitution of VRA, or VRA and PRR, did not influence the binding phenotype of mutant SU proteins (Figure 6B). By contrast, the reciprocal substitution of VRA and VRB (Figure 6C - D), or of VRB alone (Figure 7A - B) generated mutant SU proteins whose binding phenotype recapitulated the parent SU of the introduced domain. Taken together, the comparative binding assays implicate the VRB-containing substitution as the major determinant of binding phenotype, but also indicate a contribution of the VRA domain. Specifically, the substitution of both VRA and VRB from FeLV-945 into FeLV-A/61E was shown to recapitulate the FeLV-945 binding phenotype (Figure 6C) but the substitution of VRB alone does not (Figure 7A), thus suggesting a role for VRA in conferring the full activity. The effect of VRA on determining binding phenotype is apparently dependent on interaction with VRB, since substitution of FeLV-945 VRA into FeLV-A/61E did not affect binding in the absence of FeLV-945 VRB (Figure 6B). The 124-amino acid VRA-containing substitution constructed for these studies includes eight amino acid sequence differences between FeLV-945 and FeLV-A/61E, seven of which are localized within the consensus VRA domain (positions 55, 58, 60, 63, 69, 98, 99; Figure 4A). All of these substitutions are shared among cohort isolates, and five of them represent the residues identified by computational modeling as potential contributors to binding phenotype (Figure 5). However, substitution of the FeLV-945 sequence into FeLV-A/61E at all five positions failed to recapitulate the FeLV-945 binding phenotype, consistent with the observation that replacement of the entire VRA domain from FeLV-945 into FeLV-A/61E did not influence binding in the absence of VRB substitution. These results indicate that FeLV-945 VRB can positively impact SU binding in the context of VRA from either FeLV-A/61E or FeLV-945, but the optimal binding interaction is achieved in the presence of FeLV-945 VRA.

Within the VRB-containing segment implicated as the major determinant of binding phenotype are eight amino acid sequence differences between FeLV-945 and FeLV-A/61E, three of which (positions 143, 147, and 149) are localized within consensus VRB. Mutational analysis, however, demonstrated that these residues could not confer the enhanced binding phenotype of FeLV-945 VRB. Mutational analysis of four other residues (positions 128, 130, 156, 164) that neighbor consensus VRB and are shared among other cohort isolates similarly demonstrated that these do not influence relative binding affinity. Indeed, only when the isoleucine-to-valine change at position 186 was incorporated into the mutants was the binding phenotype affected (Table 1), and point mutation implicated the single valine residue at position 186 in FeLV-945 SU as the major determinant of increased binding affinity (Figure 8). Computational modeling sheds light on the mechanism by which valine 186 as represented in FeLV-945 may influence increased binding affinity as compared to isoleucine 186 as represented in FeLV-A/61E (Figure 9). Acting through an effect on position of the neighboring residue Q110, the relatively bulky isoleucine side chain at position 186 results in a narrowing of the predicted receptor-binding cleft at its lower end (Figure 9A). By contrast, the relatively compact valine side chain at position 186 allows Q110 to assume an orientation out of the receptor-binding cleft thus widening the lower end approximately two-fold (Figure 9B). The impact on conformation of the receptor-binding cleft is then predicted to influence the relative binding affinity for FeLV-A receptor. This influence of valine 186 is apparently further influenced by residues in VRA that make contact with receptor, since a more optimal binding phenotype is observed in the presence of VRA from FeLV-945 as compared to that of FeLV-A/61E. This may explain why FeLV-922 and FeLV-1046 SU proteins showed an even greater binding affinity than FeLV-945, since both contain valine 186 and additional sequence differences within VRA that are not shared between the two or with other cohort isolates (Figure 4). This hypothesis will be further examined in future studies. While several studies have shown the VRA domain to be sufficient for infection, efficient infection is known to require the VRB domain as well [15-21]. The present findings implicate a single residue in the influence of VRB on FeLV-945 SU binding affinity, and suggest a mechanism by which that residue interacts with the VRA domain to effect its influence.

## Conclusions

The SU protein of FeLV-945, a unique natural isolate of feline leukemia virus, directs the induction of non-T-cell multicentric lymphoma through an as yet unknown mechanism. FeLV-945 SU is shown here to bind its cell surface receptor, feTHTR1, with significantly greater efficiency than does prototype FeLV-A SU when present on the surface of virus particles or in soluble form. The increased binding activity demonstrates a 2-fold difference in K_d _relative to prototype FeLV-A SU. Computational modeling and mutational analyses implicate a region containing the VRB domain of FeLV-945 SU as the major determinant of increased binding affinity, although a region containing the VRA domain appears to play a role. The results implicate a single residue adjacent to consensus VRB, valine 186, as the major determinant of increased binding affinity. Computational modeling suggests a molecular mechanism by which residue 186 interacts with residue Q110 to effect increased binding affinity.

## Methods

### Cells and viruses

Feline 3201 cells, an FeLV-negative T-lymphoid line derived from a natural thymic lymphoma, were maintained in a medium of 50% Leibovitz L-15 and 50% RPMI 1640 with 15% fetal bovine serum (FBS). FEA cells, a continuous line of feline embryonic fibroblasts, were maintained in Eagle's minimum essential medium with 10% FBS and nonessential amino acids. AH927, a continuous line of feline embryonic fibroblasts, was maintained in Dulbecco's Modified Eagle Medium (DMEM) with 10% FBS. Murine MDTF/H2 cells, a gift of Dr. Julie Overbaugh, represent a continuous line of *Mus dunni *tail fibroblasts engineered to express the FeLV-A receptor, feTHTR1, and were maintained in DMEM with 10% FBS and 0.6 mg/ml geneticin [25]. Human embryonic kidney 293T/17 cells (ATCC CRL-11268) were maintained in DMEM with 10% FBS. Molecularly cloned, infectious proviral DNAs used in the study include FeLV-A/61E [27] and the infectious recombinant viruses 61E/945L and 61E/945SL. As described previously, 61E/945L was generated by replacement of the FeLV-A/61E LTR with that of FeLV-945 and 61E/945SL was generated by replacement of the FeLV-A/61E LTR and SU gene with corresponding sequences from FeLV-945 [6,8]. Pseudoptyped virus particles bearing envelope proteins of FeLV-945, FeLV-922, FeLV-1049, FeLV-1306, or FeLV-1046 were constructed as previously described [6].

### SU expression constructs

CS2-FeLV-A-61E-SU-HA [13], a gift of Dr. Julie Overbaugh, encodes the FeLV-A/61E SU gene corresponding to amino acids 1 to 435, fused at the carboxy terminus to a hemagglutinin (HA) epitope tag. CS2-FeLV-945-SU-HA was generated by replacing a *MscI *to *NcoI *fragment (basepair 100 to 1214) of the SU gene in CS2-FeLV-61E-SU-HA with the corresponding fragment from FeLV-945 [GenBank:AY662447]. An empty vector control, designated pCS2/Ctrl, was generated by religation of BamHI-digested CS2-FeLV-A-61E-SU-HA. 61E/945-VRA and 61E/945-VRA/VRB were generated by replacing a *HindIII*-to-*HindIII *fragment or an *NdeI*-to-*Bsu36I *fragment of CS2-FeLV-A-61E-SU-HA, respectively, with the corresponding fragment of CS2-FeLV-945-SU-HA. 61E/945-VRA/PRR was generated by replacing a *Bsu36I*-to-*NcoI *fragment of 61E/945-VRA with the corresponding fragment of CS2-FeLV-945-SU-HA. 945/61E-VRA/VRB was generated by replacing a *NdeI*-to-*Bsu36I *fragment of CS2-FeLV-945-SU-HA with the corresponding fragment of CS2-FeLV-61E-SU-HA. 61E/945-VRB was generated by replacing a *HindIII*-to-*HindIII *fragment of 61E/945-VRA/VRB with the corresponding fragment of CS2-FeLV-61E-SU-HA. A reciprocal mutant, 945/61E-VRB, was made by replacing a *HindIII*-to-*HindIII *fragment of 945/61E-VRA/VRB with the corresponding fragment of CS2-FeLV-945-SU-HA. SU genes bearing point mutations were generated by site-directed mutagenesis (Stratagene, Agilent Technologies, Santa Clara, CA) using CS2-FeLV-61E-SU-HA as template and using the indicated primers where the introduced mutation in each case is underlined: 1) the mutant designated 61E/945-5 using primer 5'-ACTAGTGTTGGATCCTAACAACGTTCGGCATGGAGCTAGGTATAGCAGTAGCAAATATGGATGTAAAACTACAGATAG-3', 2) the mutant designated VRB3aa using primer 5'-GAGGGAGTAATCAGGACAATAGCTGCACAGGAAAATGCAACCCCC-3', 3) the mutant designated N147S using primer 5'-GGGAGTAGTCAGGACAATAGCTGTGAGGG-3', 4) the mutant designated K128N/S130T using primer 5'-GCAACCCCCTAGTCTTACAGTTCACCCAGAAGGGAAGACAAGCCTCTTGG-3', 5) the mutant designated I156V/K164R using primer 5'-GGAGAAGCTTGGTGGAATCCCACCTCCTCATGG-3', and 6) the mutant designated I186V using primer 5'-GGATATGACCCTGTCGCTTTATTCACGGTGTCCCGGCAGG-3'. The mutant designated V186I was generated using CS2-FeLV-945-SU-HA as template and using primer 5'-GGATATGACCCTATCGCCTTATTCACGGTATCCCGGCAGG-3'. The mutants designated K128N/S130T/I186V and I156V/K164R/I186V were generated using primer 5'-GGATATGACCCTGTCGCTTTATTCACGGTGTCCCGGCAGG-3' and using K128N/S130T or I156V/K164R as template, respectively.

### Generation and titering of soluble SU proteins

293T/17 cells were transfected with SU expression plasmids using Lipofectamine LTX reagent (Invitrogen Corp., Carlsbad, CA) as per the manufacturer's instructions. Approximately 48 hours after transfection, SU-containing cell supernatants were collected and filtered to remove cellular debris. Cell-free supernatants were mixed with Laemmli Buffer (Bio-Rad Laboratories, Hercules, CA) and 2.5% beta-mercaptoethanol, boiled for 5 minutes, and electrophoresed under reducing conditions in NuPAGE Novex Bis-Tris Mini Gels (Invitrogen Corp., Carlsbad, CA). Proteins were transferred to nitrocellulose and western blot analysis was performed to detect SU proteins using either the primary antibody C11D8 (Custom Monoclonals International, West Sacramento, CA) or mouse anti-HA (Invitrogen, Camarillo, CA) and an infrared dye-conjugated secondary antibody (Li-Cor Biosciences, Lincoln, NE). SU protein preparations were thereby quantified using the Odyssey Infrared Imaging System (Li-Cor). Equivalent mass amounts of each SU protein were then visualized by western blot analysis using chemiluminescent detection (Thermo Scientific, Rockford, IL) to confirm the validity of the quantitation.

### Flow cytometric target cell binding assays

Analysis of virus binding to target cells was performed using cell-free supernatants that contained viral particles which had been titered by quantifying the 50% tissue culture infectious dose (TCID50). This was accomplished by using limiting dilution assays for infectivity on FEA cells as described previously [9]. For binding assays, 4 × 10^6 ^TCID50 of either 61E/945L or 61E/945SL were incubated with 5 × 10^5 ^3201 cells in the presence of 8 μg/ml hexadimethrine bromide (Sigma-Aldrich, St. Louis, MO) for one hour at 37°C. Cells were washed in standard azide buffer (SAB; 1% w/vol FA Bacto buffer, 0.1% w/vol NaN3, 1% heat-inactivated fetal bovine serum) and incubated with C11D8 monoclonal antibody for 1.5 hours at 4°C to detect FeLV SU. Cells were then washed again in SAB and incubated with a goat anti-mouse Alexa 488-conjugated secondary antibody (Invitrogen Corp., Carlsbad, CA) for 30 minutes at 4°C. Virus-bound cells were analyzed on a Becton Dickson FACSCalibur flow cytometer with BD Biosciences CELLQuest Pro Software. In some cases, binding assays were performed with pseudotyped particles where 5 × 10^5 ^FFU of pseudotyped particles bearing envelope proteins of FeLV-945, FeLV-922, FeLV-1049, FeLV-1306, or FeLV-1046A [6] were incubated with 5 × 10^4 ^3201 cells and processed as described above. Binding assays were also performed using equivalent mass amounts of soluble SU proteins quantified as described above. Soluble SU proteins were incubated with 5 × 10^5 ^target cells for one hour at 37°C. Cells were washed in phosphate buffered saline containing 2% horse serum and incubated with either C11D8 antibody or mouse anti-HA antibody (Invitrogen, Camarillo, CA) for one hour at 4°C. Cells were then washed as before, incubated with goat anti-mouse Alexa 488-conjugated secondary antibody for 45 minutes at 4°C, and analyzed by flow cytometry. To determine the relative dissociation constants (K_d_), binding assays were performed on quadruplicate samples at increasing concentrations of SU protein from FeLV-A/61E or from FeLV-945. The relative K_d _was then calculated from the geometric mean fluorescence intensities obtained in three replicate assays at each concentration by nonlinear regression analysis using saturation binding kinetics equations in GraphPad Prism5.0 (GraphPad Software, Inc., La Jolla, CA) with an assumption of one site-specific binding. The relative K_d _values were used to calculate receptor occupancy using the following equation:

### Computational modelling

Based on the previously reported crystal structure of the receptor binding domain of FeLV subgroup B [28], the corresponding residues of FeLV-A/61E and FeLV-945 were submitted for computational modeling by the SwissModel program [29-31]. The binding cleft domains of FeLV-A/61E and FeLV-945 SU were modeled using the SwissModel program [29-31] and the molecular surfaces were calculated and drawn using 3D Molecule Viewer (Vector NTI, Invitrogen Corp., Carlsbad, CA).

### Statistical analysis

Statistical analysis of the data from replicate binding assays was performed using one-way ANOVA and Bonferroni post test.

## Competing interests

The authors declare that they have no competing interests.

## Authors' contributions

LSL conceived of the study, participated in study design and implementation, data analysis and interpretation and, together with LLB, drafted the manuscript. LLB developed SU exchange mutants and point mutants (except 61E/945-5), expressed and quantified soluble SU proteins, performed SU binding assays, and together with LSL, played the primary role in experimental design and interpretation. CC and PLR developed assay systems, quantified virus particles and performed virus binding assays. LMA performed the computational modeling, developed the SU point mutant 61E/945-5 and performed the saturation binding analysis. All authors read and approved the manuscript.
